# Delusion of Pregnancy in Frontotemporal Lobar Degeneration with Motor Neurone Disease (FTLD/MND)

**DOI:** 10.1155/2008/149086

**Published:** 2008-12-18

**Authors:** A. J. Larner

**Affiliations:** Cognitive Function ClinicWalton Centre for Neurology and NeurosurgeryLiverpoolUK

**Keywords:** Delusion of pregnancy, frontotemporal lobar degeneration with motor neurone disease

## Abstract

Psychotic phenomena such as delusions and hallucinations are rare in frontotemporal dementia syndromes but have recently been recognised as an early feature in some cases of frontotemporal lobar degeneration with motor neurone disease (FTLD/MND). A patient with delusion of pregnancy as an early feature of FTLD/MND is presented to illustrate the need to consider neurodegenerative disease as well as primary psychiatric disorder as the underlying cause of this striking symptom.

## 1. Introduction

Delusion of pregnancy has been documented as a symptom of psychiatric disorder, most often schizophrenia but sometimes psychotic depression, occurring not only in women of child-bearing age but also in postmenopausal women and in men [1–3]. A patient with delusion of pregnancy in the context of frontotemporal lobar degeneration with motor neurone disease (FTLD/MND) is presented.

## 2. Case report

A 42 year-old married woman was referred with a history of change in personality. She had become increasingly apathetic and indifferent over the previous 12 months, such that she was unable to continue her work as a healthcare professional or look after her 2 ½ year-old child. Her husband had taken over household chores and shopping, including supervision of the antidepressant medications which had been prescribed by her general practitioner for these symptoms, to which she made no clinical response. There was no past history of medical or psychiatric illness. In the family history, her father had died with motor neurone disease.

Using the Cambridge Behavioural Inventory (CBI) [4], a checklist of cognitive and behavioural symptoms, which was completed by the patient's husband, there was also evidence for impaired self-care (difficulty self-grooming), mood change (rapid shifts in emotions), change in dietary habits (eating the same food repeatedly), disinhibition (acting impulsively) and stereotyped and motor behaviours (following routines, hoarding, echolalia), all suggestive of a frontotemporal dementia syndrome.

In addition, the patient repeatedly began to state that she was pregnant. When questioned about this in the clinic, she reported having her period at the time but nevertheless still thought the possibility of pregnancy was likely, although she had not done a pregnancy test nor discussed the matter with her general practitioner. On the CBI she was noted to have "odd or bizarre

ISSN 0953-4180/08/$17.00 © 2008 – IOS Press and the authors. All rights reserved ideas that cannot be true" but no hallucinations or other delusions.

Salient findings on neurological examination were fasciculations in the tongue, wasting of shoulder girdle musculature, and pathologically brisk tendon reflexes. Cognitive testing revealed impaired scores on the Mini-Mental State Examination (22/30) and Addenbrooke's Cogntive Examination-Revised (ACE-R; 70/100). ACE-R subscores showed relatively preserved attention and orientation (14/18), memory (23/26) and visuospatial skills (13/16), but impaired verbal fluency (1/14) and language skills (19/26). Structural neuroimaging (CT and MRI) showed marked bilateral frontal brain atrophy, and functional neuroimaging (SPECT) showed bilateral frontal hypoperfusion. Electromyography showed active denervation changes with loss of motor units in both upper and lower limb muscles and sternocleidomastoid, consistent with an anterior horn cell disorder. Clinical and investigation findings therefore fulfilled diagnostic criteria for FTLD/MND [5]. In light of the family history of MND, genetic testing for mutations in the tau gene was undertaken but proved negative.

## 3. Discussion

Delusion of pregnancy has rarely been described in the context of neurodegenerative disorders rather than primary psychiatric conditions. This may simply be a reflection of the fact that neurodegenerative disorders occur more frequently with increasing age, although post-menopausal delusion of pregnancy is described [3]. Transient delusions, predominantly of somatic type and including delusion of pregnancy, have been reported in a 36 year-old woman with variant Creutzfeldt-Jakob disease [6] but prior reports of delusion of pregnancy in FTLD/MND have not been identified.

The frontotemporal dementias are often accompanied by non-cognitive neuropsychiatric manifestations such as apathy, disinhibition, loss of insight, transgression of social norms, emotional blunting, and repetitive and stereotyped behaviours [7]. Indeed, in a series of FTLD/MND patients reported from this centre, over two-thirds were under the care of a psychiatrist at time of diagnosis, some with provisional diagnoses of hypomania or depression, and all of whom were receiving either antidepressant or neuroleptic medications, sometimes in addition to anti-dementia drugs, suggesting that neuropsychiatric symptoms are not uncommon in this condition [8].

Psychotic symptoms including delusions and hallucinations are, however, rarely seen in frontotemporal dementias [9]. FTLD/MND may be an exception to this rule, sometimes manifesting an early psychotic phase characterised by hallucinations and delusions which may be dramatic and bizarre but transient [10]. A case of FTLD/MND presenting with De Clerambault's syndrome (erotomania) has been reported [11].

Hence, the possibility of neurodegenerative disorder in general and FTLD/MND in particular should enter the differential diagnosis in patients with delusion of pregnancy. Such cases might shed light on the pathophysiological substrates of delusions.

## References

[1] J. Kornischka and F. Schneider, Delusion of pregnancy. A case report and review of the literature, *Psychopathology* **36** (2003), 276–278.

[2] A.M. Adityanjee, Delusion of pregnancy in males; a case report and literature review, *Psychopathology* **28** (1995), 307–311.

[3] R.F. Harland and N.J. Warner, Delusions of pregnancy in the elderly, *Int J Geriatr Psychiatry* **12** (1997), 115–117.

[4] C. Wedderburn, H. Wear, J. Brown, S.J. Mason, R.A. Barker, J. Hodges and C. Williams-Gray, The utility of the Cambridge Behavioural Inventory in neurodegenerative disease, *J Neurol Neurosurg Psychiatry* **79** (2008), 500–503.

[5] G.M. McKhann, M.S. Albert, M. Grossman, B. Miller, D. Dickson and J.Q. Trojanowski, Clinical and pathological diagnosis of frontotemporal dementia. Report of the Work Group on Frontotemporal Dementia and Pick's disease, *Arch Neurol* **58** (2001), 1803–1809.

[6] A. Dervaux, S. Vicart, F. Lopes and M.H. Le Borgne, Manifestations psychiatriques du nouveau variant de la maladie de Creutzfeldt-Jakob. A propos d'un cas, *Encephale* **27** (2001), 194–197.

[7] M.F. Mendez, E.C. Lauterbach and S.M. Sampson, ANPA Committee on Research, An evidence-based review of the psychopathology of frontotemporal dementia: a report of the ANPA Committee on Research, *J Neuropsychiatry Clin Neurosci* **20** (2008), 130–149.

[8] S. Sathasivam, M. Doran and A.J. Larner, Frontotemporal lobar degeneration with motor neurone disease (FTLD/MND): presentations in psychiatric practice, *Int J Psych Clin Pract* **12** (2008), 138–141.

[9] M.F. Mendez, J.S. Shapira, R.J. Woods, E.A. Licht and R.E. Saul, Psychotic symptoms in frontotemporal dementia: prevalence and review, *Dement Geriatr Cogn Disord* **25** (2008), 206–211.

[10] T.H. Bak, Overlap syndromes, in: *Frontotemporal dementia syndromes*, J.R. Hodges, ed., Cambridge University Press, Cambridge, 2007, pp. 80–101.

[11] C. Olojugba, R. de Silva, L.D. Kartsounis, L. Royan and J. Carter, De Clerambault's syndrome (erotomania) as a presenting feature of fronto-temporal dementia and motor neurone disease (FTD-MND), *Behav Neurol* **18** (2007), 193–195.
